# Functional Optical Coherence Tomography for Intrinsic Signal Optoretinography: Recent Developments and Deployment Challenges

**DOI:** 10.3389/fmed.2022.864824

**Published:** 2022-04-04

**Authors:** Tae-Hoon Kim, Guangying Ma, Taeyoon Son, Xincheng Yao

**Affiliations:** ^1^Richard and Loan Hill Department of Biomedical Engineering, University of Illinois at Chicago, Chicago, IL, United States; ^2^Department of Ophthalmology and Visual Sciences, University of Illinois at Chicago, Chicago, IL, United States

**Keywords:** optoretinography, intrinsic optical signal imaging, retina, phototransduction, optical coherence tomography, retinography, functional retinal imaging, photoreceptor

## Abstract

Intrinsic optical signal (IOS) imaging of the retina, also termed as optoretinogram or optoretinography (ORG), promises a non-invasive method for the objective assessment of retinal function. By providing the unparalleled capability to differentiate individual retinal layers, functional optical coherence tomography (OCT) has been actively investigated for intrinsic signal ORG measurements. However, clinical deployment of functional OCT for quantitative ORG is still challenging due to the lack of a standardized imaging protocol and the complication of IOS sources and mechanisms. This article aims to summarize recent developments of functional OCT for ORG measurement, OCT intensity- and phase-based IOS processing. Technical challenges and perspectives of quantitative IOS analysis and ORG interpretations are discussed.

## Introduction

The retina is a neurovascular network complex that can be frequently affected by eye diseases such as age-related macular degeneration (AMD), diabetic retinopathy (DR), glaucoma, and inherited retinal dystrophies (IRDs). Optical imaging methods, such as light fundus photography and fluorescein angiography (1–3), can reveal morphological abnormalities for eye disease diagnosis and treatment assessment. Scanning laser ophthalmoscopy (SLO) (4, 5) can provide improved spatial resolution and image contrast. Optical coherence tomography (OCT) (6, 7) can provide depth-resolved, cross-sectional images of individual retinal neural layers. As one special OCT modality, OCT angiography (OCTA) (8–11) can enhance the visibility of individual retinal capillary plexuses. Adaptive optics (AO) can be incorporated to enhance the resolution of the fundus camera (12, 13), SLO (14, 15), and OCT (16–18). These methods for morphological imaging of the retina provide vital information for clinical management of eye diseases.

However, retinal diseases are often quite advanced before they draw clinical attention, by which time the retina may be functionally abnormal. Structural and functional abnormalities in the retina are often not correlated in the spatial location and time window. Therefore, an objective method for functional assessment of the retina promises early detection and longitudinal therapeutic assessment of retinal degenerative diseases. Electroretinography (ERG) and multifocal ERG (19, 20) can objectively assess retinal physiological function. However, separate morphological imaging and functional measurement can be costly and time-consuming. Moreover, different spatial resolutions of morphological imaging and functional measurement may challenge clinical evaluation.

Intrinsic optical signal (IOS) imaging of the retina (21–29), also termed as optophysiology (30), optoretinogram (31–35), or optoretinography (25, 36–40) (ORG), promises a non-invasive method for objective assessment of retinal physiological function. The terminology ORG is an analogy to ERG. ERG is based on the electrical measurement of stimulus-evoked electrophysiological activities, while ORG refers to IOS imaging of corresponding light property changes in the retina due to functional activity. Time-lapse light microscopy and fundus camera have been used for two-dimensional IOS imaging study of isolated retinal tissues and intact eyes (21, 22, 28). By providing the unparalleled capability to differentiate individual layers of the retina, OCT has been actively used for IOS imaging of animal and human retinas (25, 29, 31–45). OCT is an interferometric imaging technique that acquires interference fringe patterns generated by the superposition of back-scattered lights from the sample and reference arms. The Fourier transform of the fringe patterns provides intensity information on the scattering object and allows access to information about the axial position of the scattering object within the retina. Thus, intensity and phase information has been utilized in quantifying the stimulus-evoked IOS in the retina. However, clinical deployment of the OCT-based ORG is still challenging due to the lack of a standardized imaging protocol and the complication of signal sources and physiological mechanisms. In the following sections, we will summarize recent developments of functional OCT systems for IOS imaging, OCT intensity and phase-based processing for quantitative IOS analysis. Technical challenges and perspectives of quantitative ORG measurement and interpretation will be discussed.

## Functional OCT Developments for Intrinsic Signal ORG

The retina is thin, transparent, and stratified into distinct cellular layers. Since the stimulus-evoked retinal activity was found to alter intrinsic optical properties at different layers, OCT has been actively investigated for depth-resolved IOS imaging (22, 34). Both time-domain and Fourier-domain OCT systems have been demonstrated for IOS imaging. Time-domain OCT was first demonstrated for depth-resolved observation of IOS in the freshly isolated frog (29) and rabbit (30) retinas. Fourier-domain OCT was later employed to validate *in vivo* IOS imaging of intact rat eyes (46). A hybrid line-scan SLO and OCT system was also employed for confocal-OCT IOS imaging study of isolated retinas (47). Given the improved imaging speed, Fourier-domain OCT has dominated recent IOS imaging studies of both animal (33, 35, 37, 39, 42, 44, 48–52) and human (25, 31, 32, 36, 37, 53–55) retinas.

**Figure 1:**
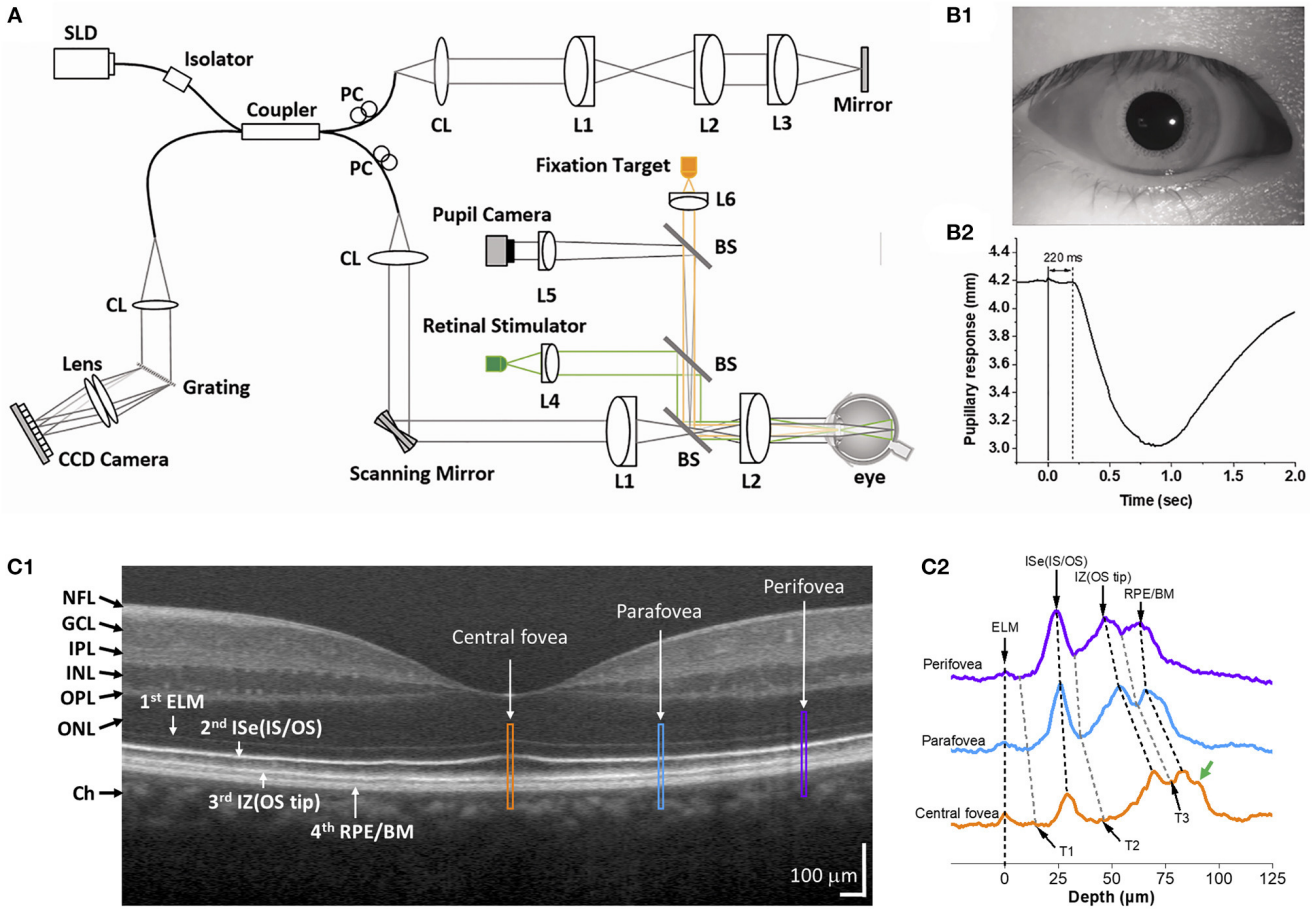
**(A)** Representative functional OCT system for IOS/ORG imaging. BS, beam splitter; CL, collimation lens; Lenses, L1, L2, L3, L4, L5, and L6; PC, polarization controller; SLD, superluminescent diode. **(B)** Representative pupil image **(B1)** and pupillary response **(B2)**. Reprinted with permission from Son et al. (25). **(C1)** Representative OCT B-scan of the human retina and **(C2)** reflectance profiles of the central fovea (yellow), parafovea (blue), and perifovea (purple). Yellow, blue, and purple windows in **(C1)** indicate retinal regions for comparative reflectance profile analysis. The green arrow in **(C2)** indicates a possible band, i.e., Bruch's membrane. T1–T3 indicate trough positions along the reflectance profiles. NFL, nerve fiber layer; GCL, ganglion cell layer; IPL, inner plexiform layer; INL, inner nuclear layer; OPL, outer plexiform layer; ONL, outer nuclear layer; ELM, external limiting membrane; ISe, inner segment ellipsoid; OS, outer segment; IZ, interdigitation zone; RPE, retinal pigment epithelium; BM, Bruch's membrane. Modified with permission from Yao et al. (56).

Figure 1 shows an exemplary Fourier-domain OCT system used for IOS imaging of the human retina. The system is a point-scan spectral-domain OCT, commonly used for clinical and research purposes. A fixation target is used to minimize eye movements during imaging. The system consists of two light sources, one near infrared superluminescent diode (SLD) for OCT imaging, and a visible light source to produce retinal stimulation. The OCT probe beam is raster-scanned over the retinal tissue. A pupil camera helps to align the OCT probe beam for optimal light incidence through the pupil (Figure 1B1) and to measure the time course of pupillary light response (Figure 1B2). The recording speed is 100 B-scan/s at a 70 kHz A-scan rate. Point-scan OCT benefits from the confocal aperture of the single-mode fiber that rejects multiply scattered light. However, imaging speed is limited for volumetric data acquisition.

Line-scan (40) or full-field (57) OCT can significantly improve the imaging speed by the parallel acquisition of lateral and axial information. Full-field OCT allows imaging without lateral phase noise by employing a collimated illumination over the retinal area with detection by a 2D camera. High-speed 3D imaging can reduce intraframe eye movement artifacts, which permits robust registration of frames and tracking of photoreceptors, returning stable OCT phase information. Access to stable phase information allows detecting cellular deformations much smaller than its axial resolution, and the phase information has been recently used to measure light-evoked photoreceptor outer segment (OS) deformation (32, 36, 55, 58). However, the parallel OCT suffers resolution loss from multiple scattering crosstalk. In addition, a tradeoff for increasing imaging speed is a reduction in the imaging area.

AO can be incorporated to improve further the OCT spatial resolution (36, 40). The AO subsystem generally consists of three elements, including the wavefront sensor, the deformable mirror, and the control computer to dynamically measure and correct low- and high-order wavefront aberrations of the eye. The current state-of-art AO-OCT system has the resolution to reveal the 3D reflectance profile of individual cone photoreceptors and provide sufficient sensitivity to detect light-evoked optical path length (OPL) changes as small as 5 nm in the individual cones (36). Azimipour et al. (31) further demonstrated a combined AO-SLO-OCT for ORG measurement of rod and cone photoreceptors in the human retina. The SLO was utilized to guide the type and location of photoreceptors in the OCT volume. However, clinical deployment of AO-OCT is still challenging due to technical factors such as high cost, optical complexity, system size, data volume, and image postprocessing (59).

## OCT Data Processing for Intrinsic Signal ORG

Both intensity and phase-based processing methods have been developed to quantify the stimulus-evoked IOS in the retina.

### OCT Intensity-Based IOS Processing

The OCT intensity-based IOS processing can be divided into two categories: OCT brightness change and OCT band analysis. The intensity-based processing takes advantage of the fact that morphological deformation of the retinal neurons can directly affect IOS, and physiological activities in the retinal neurons and vasculature can cause local variation of optical properties, such as refractive index, scattering, reflection, or birefringence.

#### OCT Brightness Analysis

**Figure 2:**
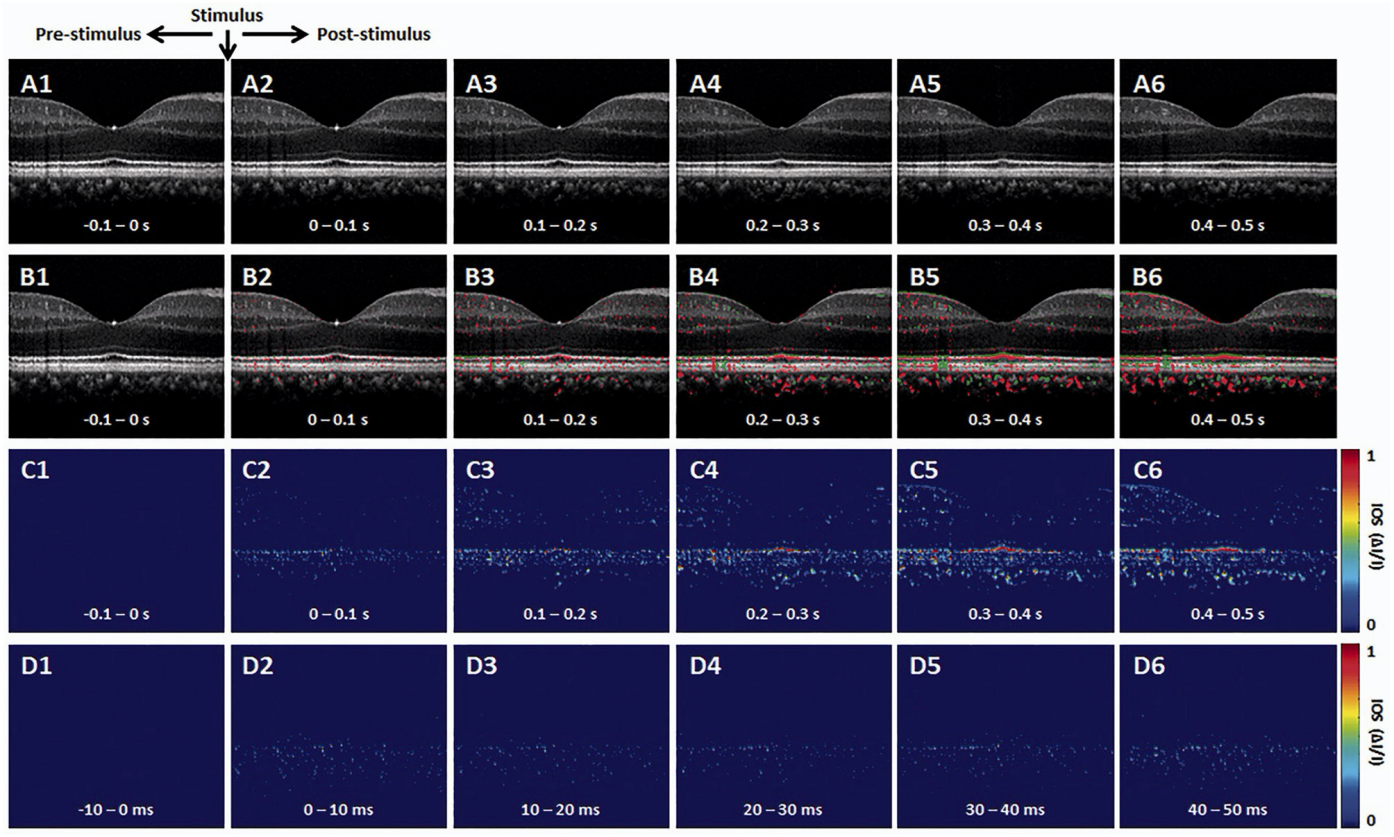
Representative OCT intensity-based IOS imaging. **(A)** Representative OCT image sequence. **(B)** Corresponding IOS distributions of positive (red) and negative (green) changes. **(C)** IOS magnitude sequence with 0.1 s time intervals. **(D)** IOS magnitude sequence with 10 ms time intervals. Reprinted with permission from Son et al. (25).

The OCT brightness change analysis was devised to detect local variations in pixel intensity value due to light stimulus within the retina. Previous studies using brightness change analysis detected localized IOS change both in the inner and outer retina (42, 51, 60–62). Figure 2 illustrates representative time-lapse OCT recording and a result from the brightness change analysis (63). The data processing is described as follows (42, 62, 64). First, raw OCT B-scans need to be registered to compensate for eye movements by a sub-pixel registration algorithm. The intensity of each pixel can then be normalized based on the inner retinal intensity to limit the effect of pupillary response (25). Next, from a sequence of the registered B-scans, the “active” IOS pixels are identified. Any pixel that significantly changes its intensity value after the light stimulus is identified as the active-IOS pixel, which can be either positive (intensity increased) or negative sign (intensity decreased). The number of active IOS pixels can be quantified for comparative study (39, 64). In addition, the intensity value of active IOS pixels can be traced over time after subtracting the background pixel intensity value from pre-stimulus B-scans (61, 63). Figure 2 demonstrates that light-evoked positive (red) and negative (green) IOSs were observed in the human retina. As shown in Figures 2B–D, the fast IOS was promptly observed after stimulus onset and primarily confined within the photoreceptor region. Since ~220 ms time window was available without pupillary response (Figure 1B2), it would be feasible to monitor the fast photoreceptor-IOS in non-mydriatic conditions (25). The brightness analysis has been demonstrated for robust detection of transient photoreceptor response; however, further investigation is needed to scrutinize IOS origin from the inner retina.

#### OCT Band Analysis

The retina is stratified into multiple-layered structures. OCT can probe the axial position of each neural and synaptic layer and visualize these layers as hyper- and hypo-reflective bands. Measuring the band alterations under different light conditions is one of the critical parameters in ORG measurement (35, 54, 65–67). There is a growing interest, especially in assessing outer retinal bands, such as photoreceptor inner segment (IS), OS, and subretinal space (SRS), and retinal pigment epithelium (RPE).

**Figure 3:**
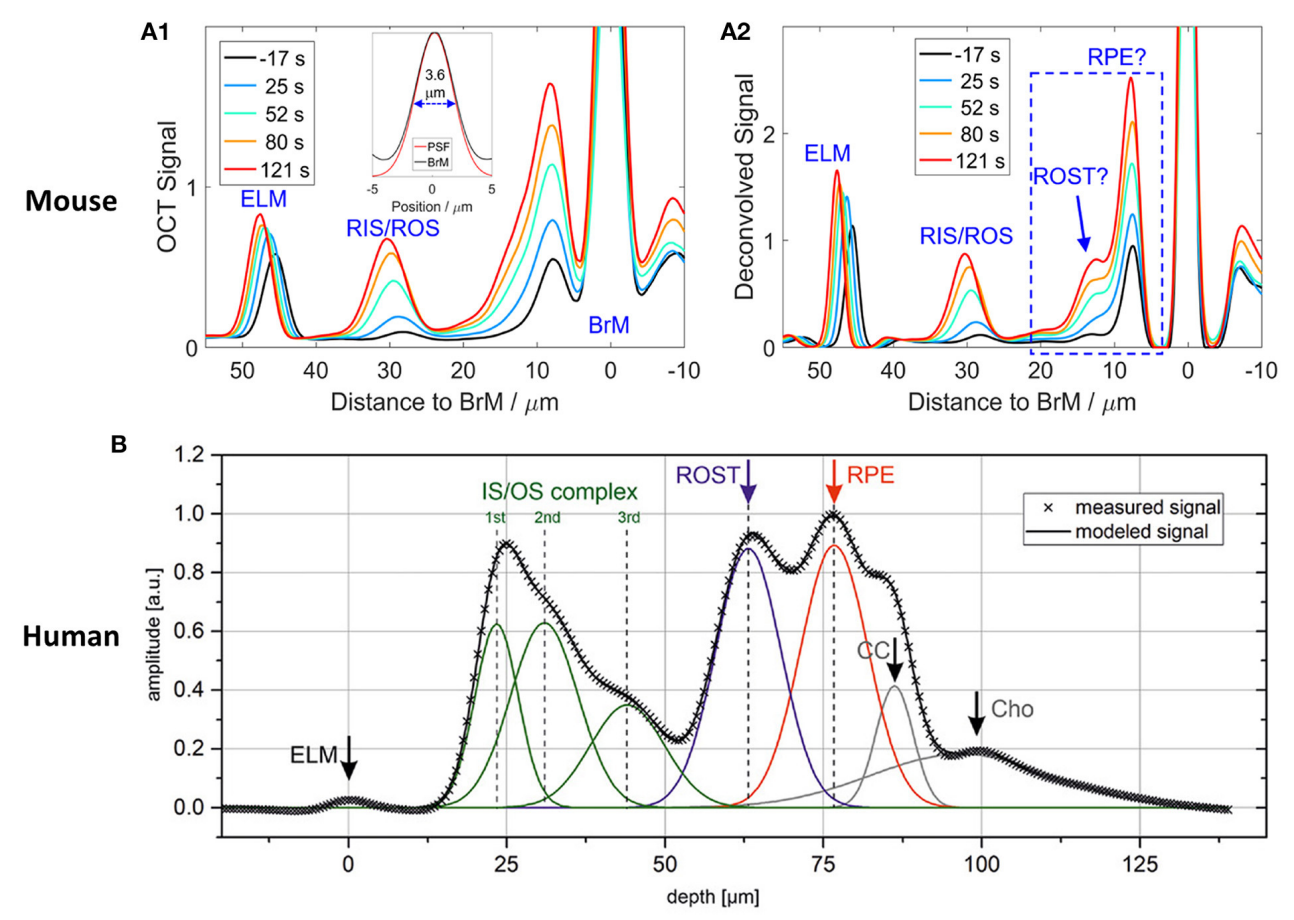
Representative OCT hyper-reflective band analysis. **(A)** Deconvolution method. **(A1)** Depth scattering profiles of the retina of an albino mouse. **(A2)** Deconvolution analysis reveals that the backscatter band nearest to Bruch's membrane (BrM) on the anterior side comprises two distinct components (question marks are used to indicate that the assignment to structures required confirmation). Reprinted with permission from Zhang et al. (35). **(B)** A-line signal modeling by summation of seven Gaussian curves. Gray crosses represent the measured data points, and the black line is the summation of the individual model curves (green, blue, red, and gray lines). ELM, external limiting membrane; IS/OS, inner segment/outer segment complex; ROST, rod outer segment tips; RPE, retinal pigment epithelium; CC, choriocapillaris; Cho, choroid. Reprinted with permission from Messner et al. (54).

Figure 3 illustrates outer retinal bands and analysis methods. Zhang et al. demonstrated a deconvolution method for band analysis in the mouse retina (Figure 3A). OCT spectra were initially Fourier transformed after four times zero-padding, and the resultant A-scan profiles were remapped onto a linear scale. Next, they used deconvolution to extract additional information from the hyperreflective bands. Specifically, the averaged A-line profiles were deconvolved with the MATLAB “deconvlucy” function, and the hyperreflective bands from each time point fitted with Gaussian functions, providing three parameters (position, amplitude, and full width at half maximum) that can be used for OCT band analysis. This method was used to measure the length of photoreceptor OS over the diurnal cycle in albino mice (35). Messner et al. demonstrated modeling the OCT A-line profiles by fitting normal distribution curves and observing their position changes over time in the human retina (Figure 3B). To better determine the position of outer retinal bands, a signal model for the A-scan averages was developed in the software OriginPro 2019b using the “multiple peak fitting” function. The signal model for the A-scan average provided quantitative parameters by tracking the position of the peaks attributed to the boundaries of the outer retinal layers during baseline and stimulation conditions (54). In addition, Kim et al. recently demonstrated transient band shifting during the initial dark adaptation period in the mouse retina. The high-speed imaging recorded repeated B-scans at the same retinal plane for 5-min dark-adaptation. The volumetric average was conducted for OCT A-line band analysis, and linear interpolation was employed to enhance the sampling density of the average A-line band analysis (65). Yao et al. suggested a more detailed band analysis by accounting for not only hyper-reflective band location but also relative distances between hyper- and hypo-reflective bands to better establish the correlation of each band to the outer retina structure (Figure 1C2). This approach may provide additional insight into the outer retinal structure and its dynamics under different light conditions (56). The band analysis requires clear boundary information, and better axial resolution can resolve more detailed morphological alterations. It should also be noted that the band composition is different depending on the eccentricity (56), and different normalization methods can directly impact outputs.

### OCT Phase-Based Processing

Evaluation of the phase of interference fringes allows access to information about OPL changes. Given a pair of clear hyper-reflective bands resolved within the retina, the recent development of phase-resolved OCT can offer sensitivities to photoreceptor OS deformation on a nanometer scale, much smaller than the axial resolution of the OCT system.

#### OCT Band Boundary Measurements for Optical Path Length Estimation

**Figure 4:**
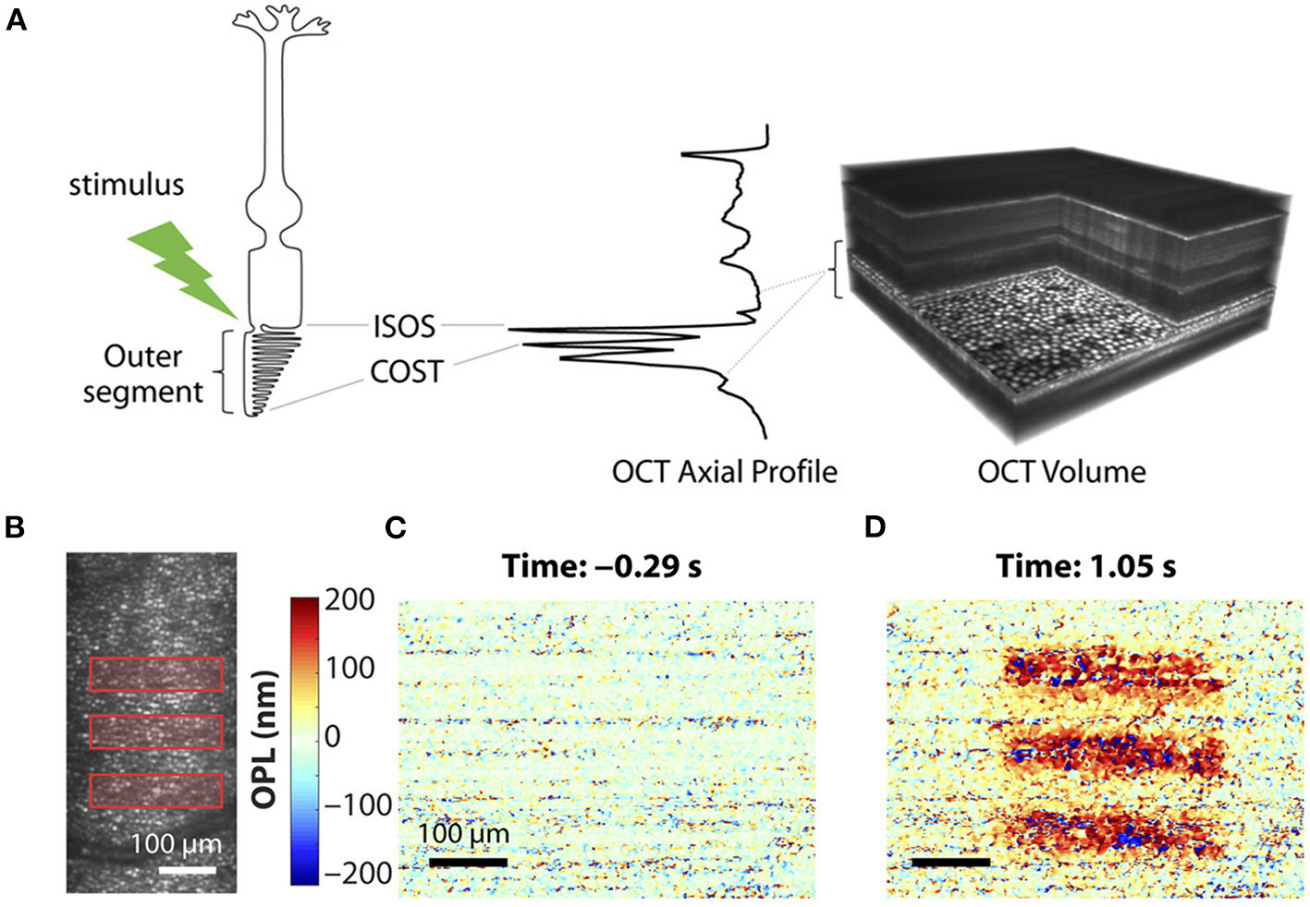
Phase-resolved OCT imaging for optical path length (OPL) estimation. **(A)** Optoretinography experimental paradigm. A three-dimensional (3D) OCT volume with AO allows resolving the cone mosaic in an en face projection and the outer retinal layers in an axial profile corresponding to the ISOS and COST. Stimulus (528 ± 20 nm, green)–driven changes in a cone photoreceptor are accessible by computing the time-varying phase difference between the proximal and distal OCT reflections encasing the outer segment. **(B)** Optoretinography reveals functional activity in cone outer segments. Illumination pattern (three bars) drawn to scale over the line-scan ophthalmoscopic image. **(C,D)** The spatial map of OPL changes between the ISOS and COST before **(C)** and after stimulus **(D)**, measured at 20-Hz volume rate. Reprinted with permission from Pandiyan et al. (32).

Given the premise that the photoreceptor OSs change in length by light stimulus, OCT phase information has been used to estimate the OPL change of photoreceptor OSs. The photoreceptor OS is long and narrow; thus, it behaves like an optical waveguide (68). In addition, there are relatively strong reflections from each end of the photoreceptor (IS/OS junction and OS tip), well suited for OPL estimation using hyper-reflective band positions. Recent advances in parallel OCT and incorporated AO subsystem enable the measurement of stable phase information. Since the phase in a single layer does not carry information to evaluate the length change of the photoreceptors, it is necessary to compare two phases between two different retinal layers and between two different time points. Figure 4 demonstrates phase-resolved OCT imaging for OPL estimation. The data processing is described as follows (55). First, the recorded OCT volumes were reconstructed, and each pixel of each reconstructed volume was then referenced to the respective co-registered pixel in one specific volume. Next, the retinal layers carrying the information about the OS length need to be segmented. Two layers used for segmentation are generally photoreceptor OS tips (POST) and inner-outer segment junction (IS/OS). In general, several axial pixels are averaged centered around the peak point of each layer. The temporal evolution of optical phase difference is then computed between the POST and IS/OS (Φ_POST_ - Φ_ISOS_) to yield a measure of light-induced relative phase changes between POST and IS/OS. The phase difference at the two layers is then converted to OPL using the relation ΔOPL = (λ_c_/4π) × (Φ_COST_ - Φ_ISOS_), where λ_c_ = central wavelength of OCT light source. A study showed that the magnitude of these OPL changes was strongly correlated with light-induced activity, and they utilized this correlation to classify three cone classes (69). In principle, relative OPL changes between any two layers, including inner retinal layers, can be estimated (70); however, the two boundaries must present clear peak bands with a high signal-to-noise ratio. The stability of phase data is crucial in the OPL estimation and is often corrupted by ocular movements. Thus, the phase-resolved ORG measurement generally requires ultrahigh-speed recording and voxel-wise registration. It should also be noted that different shapes of cone OSs presented from the central fovea to the parafovea may affect OPL estimation (71).

#### Differential-Phase Analysis for Spatiotemporal Mapping at Pixel Resolution

**Figure 5:**
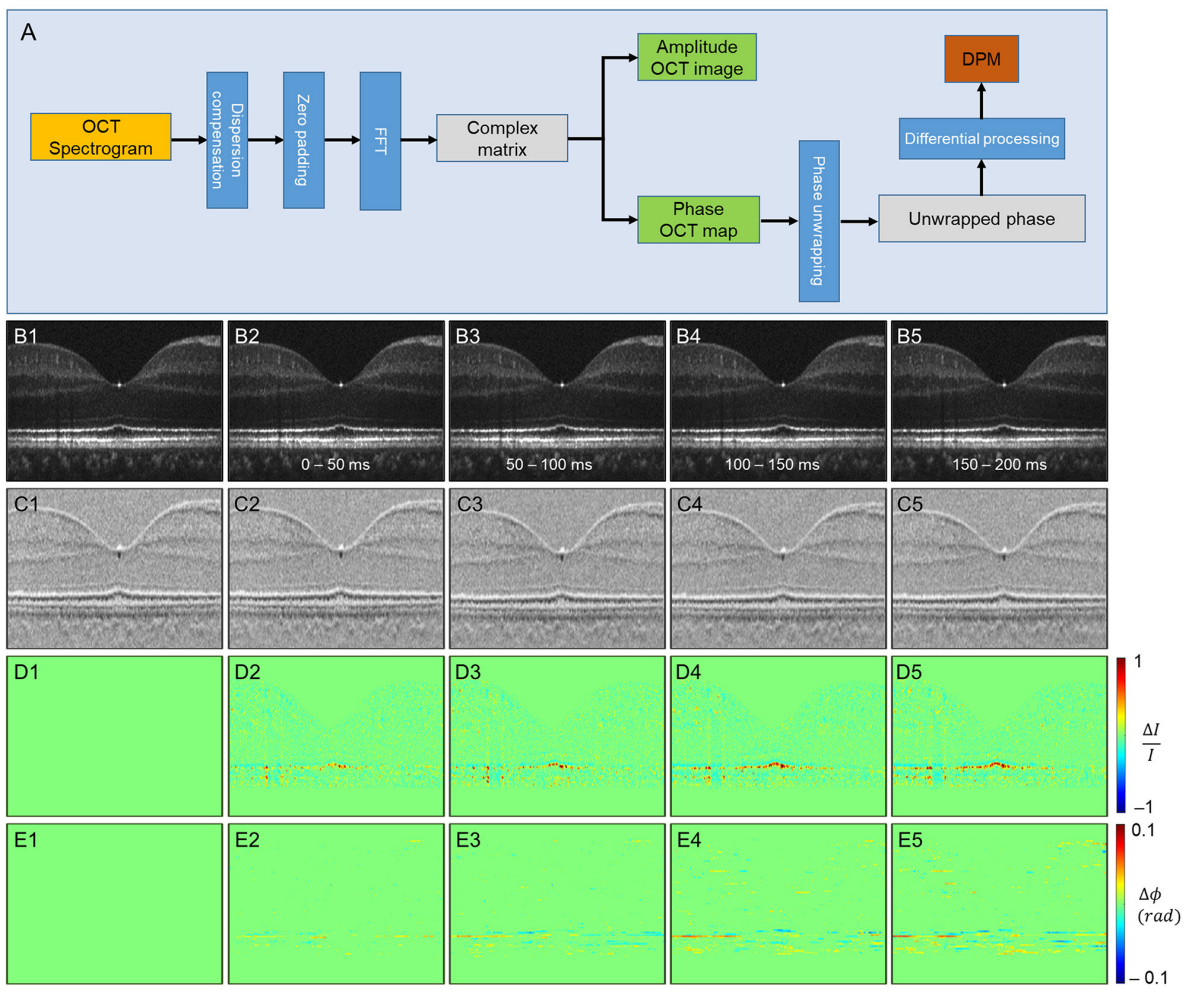
Phase-resolved OCT imaging for differential-phase mapping (DPM) analysis. **(A)** A flow chart of amplitude OCT and DPM processing. **(B–E)** The amplitude-IOS and phase-IOS distribution. **(B)** Amplitude image sequence. **(C)** DPM sequence. **(D)** Amplitude-IOS sequence. **(E)** Phase-IOS sequence. Reprinted with permission from Ma et al. (37).

Ma et al. (37) recently demonstrated a new approach that simultaneously monitors the phase changes along the whole retinal depths, called differential phase mapping (DPM). DPM was devised to analyze the spatiotemporal phase change at pixel resolution. The processing flow was as follows (Figure 5A). Digital dispersion compensation, zero-padding, and fast Fourier transform (FFT) were applied to raw data to get a complex matrix with amplitude and phase information. Then, the OCT phase was unwrapped along the A-scan direction. Finally, the unwrapped phase was differentiated along the A-scan direction. If the scatters of the adjacent pixels are both in the center, the value of the pixel in DPM is 4πnL/λ_c_, where *n* denotes the refractive index of the tissue, *L* denotes the pixel length, λ_*c*_ denotes the center wavelength of the light source, and the coefficient 4π is because the OCT measures the back-scattered light. If the distance of two scatterers is less than *L*, the pixel value of DPM will be smaller than 4πnL/λ_c_, vice versa. Therefore, the DPM represents the relative scatter distance of the sample. Compared to the OCT amplitude image (Figure 5B), DPM also reveals the structural information representing scatter locations (Figure 5C). After stimulation, both amplitude and phase IOS appeared at the outer retina layers (Figures 5D,E). The phase IOSs of different layers at different time courses indicated the depth association of phototransduction in the outer retina. Compared to conventional phase-resolved OPL measurement, computing the phase change between two selected locations, DPM shows the phase change over all the retina depths simultaneously, which could help understand the phase dynamics between retinal layers.

## ORG Interpretations and Its Challenges

ORG measurement and interpretation are challenging due to the multiple signal sources and variable OCT instruments, and experimental protocols.

### Retinal Neurovascular Coupling and Inner Retinal Response

**Figure 6:**
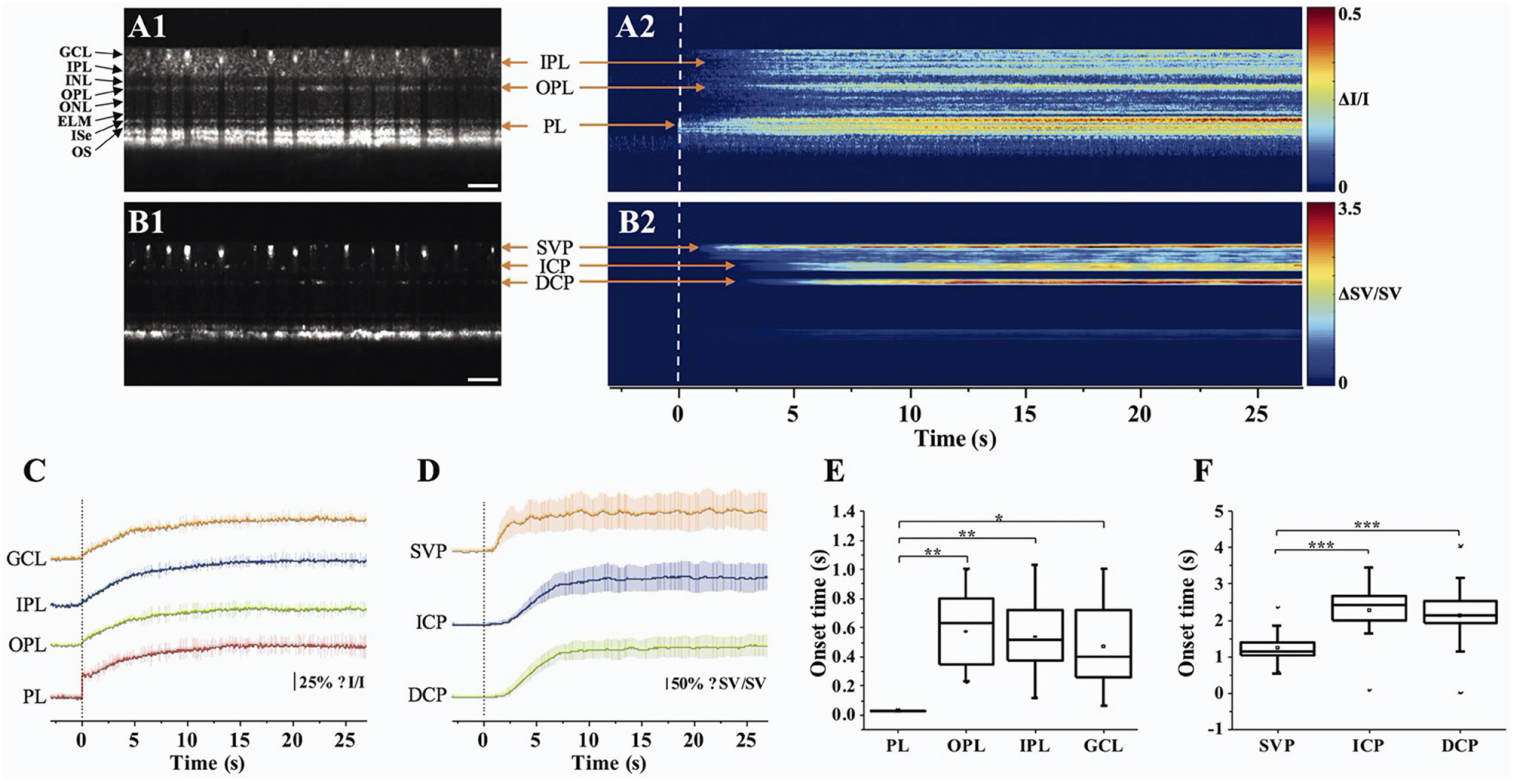
Retinal neurovascular coupling and inner retinal IOS response. **(A)** Representative flattened **(A1)** OCT B-scan and **(A2)** spatiotemporal neural-IOS map. **(B)** Representative flattened **(B1)** OCTA B-scan and **(B2)** spatiotemporal hemodynamic-IOS map. Scale bars in **(A1,B1)** indicate 500 μm. **(C)** Neural-IOS changes of photoreceptor layer (PL), outer plexiform layer (OPL), inner plexiform layer (IPL), and ganglion cell layer (GCL). **(D)** Hemodynamic-IOS changes of superficial vascular plexiform (SVP), intermediate capillary plexiform (ICP), and deep capillary plexiform (DCP). **(E)** Averaged onset times of neural-IOS changes at PL, OPL, IPL, and GCL. **(F)** Averaged onset times of hemodynamic-IOS changes of SVP, ICP, and DCP. Modified with permission from Son et al. (63). P values for statistical significances are indicated by asterisks: *P < 0.05; **P < 0.01; ***P < 0.001.

Retinal blood flow is actively regulated in response to neuronal activity (72), called neurovascular coupling. The impaired coupling mechanism is commonly associated with microvascular pathologies in the retina (73, 74). Thus, spatiotemporal mapping of transient neural activity and subsequent hemodynamic responses promises early detection of retinal diseases. Based on the intensity-based processing, stimulus-evoked IOS changes have been detected in retinal layers and the vascular network within the retina. Son et al. demonstrated concurrent mapping of neural- and hemodynamic-IOS to monitor retinal neurovascular coupling in the mouse retina (50, 63). They leveraged OCT angiography (OCTA) maps to isolate the retinal vasculature at a single capillary level resolution. The OCTA-guided IOS data processing enables two functional images: a neural-IOS map and a hemodynamic-IOS map. Flicker stimuli were used to induce a robust hemodynamic response. As shown in Figure 6, fast photoreceptor-IOS was first observed right after the stimulus onset, while hemodynamic-IOS was revealed with a significant time delay. The temporal progress of hemodynamic-IOS responses was also varied in different vascular plexuses (Figure 6D). However, the mechanism of the hemodynamic-IOS responses in large blood vessels and capillaries is not understood yet. Only a few hypotheses have been proposed, such as different neural metabolic demands in individual retinal layers, passive dilation of downstream capillaries, and mural cells' intervention on blood flow regulation (63). In addition, 2D cross-sectional imaging would be challenging for monitoring delayed hemodynamic IOS in the human retina due to motion artifacts. Moving correction is impossible if the retina moves perpendicularly to the imaging plane as there is no data to use in correction. High-speed parallel OCT would be desirable for neurovascular coupling study. Another task is to appreciate how local signal variations occur in blood vessel regions associated with flux change, hematocrit, and diameter increase.

Aside from hemodynamic information, the inner retina itself also revealed IOS change (Figure 6C). Previous mouse studies reported that a short pulse stimulation mainly induced the fast-photoreceptor IOS, while increased stimulus duration or flicker stimulation can induce IOS changes in the inner retina (41, 42, 50, 75). Although the signal source is underappreciated, it was postulated that the slow inner retinal IOS might be associated with an integral effect of electrophysiological signal transduction between multiple inner retinal neurons, such as bipolar cells, amacrine cells, horizontal cells, and ganglion cells. The plexiform layer in the retina consists of a complex synaptic network containing numerous dendrites from different types of neurons (76, 77). Their synaptic signaling might affect optical signal properties. Pfäffle et al. (70) recently showed simultaneous imaging of the activation in the photoreceptor and ganglion cell layer/inner plexiform layer (GCL/IPL) in the human retina by using phase-sensitive full-field swept-source OCT (FF-SS-OCT). Although the signals from the GCL/IPL were 10-fold smaller than those from the photoreceptor, GCL/IPL signals were still detectable with suppression of motion artifacts and blood flow pulsations in the retinal vessels. The phase difference of the GCL and the IPL was calculated to evaluate the light-evoked OPL changes, and they found that the OPL between GCL and IPL increased about 40 nm in the stimulated area, and the increase in OPL reached its maximum of about 40 nm after approximately 5 s. However, the mechanism of inner retinal IOS changes from both intensity-based and phase-based results is poorly understood. In addition, retinal vasculature is embedded in the inner retina; thus, blood flow pulsation and inhomogeneous intensity distribution may complicate signal interpretations.

### Transient Deformations of Photoreceptor Outer Segment

As the center of phototransduction, retinal photoreceptors are responsible for converting photon energy to bioelectric signals for following vision processing in the retina. Retinal photoreceptors are the primary target cell of retinal degenerative diseases such as AMD and retinitis pigmentosa (RP); thus, non-invasive monitoring of functional integrity of photoreceptors is of great interest. The photoreceptors are the most well studied among retinal cell types by intrinsic signal ORG measurement as they exhibit an exceptionally reproducible light-driven response.

At first, time-lapse near-infrared light microscopy was used to image transient IOS changes in freshly isolated retinas, and it was found that the IOS rapidly occurred in the photoreceptor cells after the visible light stimulus (78, 79). The magnitude and time course of IOS changes were found to be correlated with the stimulus strength (78). In addition, transient shrinkage-induced deformation in photoreceptors was directly observed in both amphibian (80) and mammalian (81) retinas. The shrinkage-induced deformation was mainly observed in the photoreceptor OSs that rapidly shifted toward the direction of the visible light stimulus (47). It turned out that the onset time of the photoreceptor shrinkage was almost identical to that of photoreceptor-IOS change, suggesting that the OS conformational change should correlate with the phototransduction process. A hybrid confocal-OCT study further demonstrated that the photoreceptor OS is the anatomic source of the transient photoreceptor deformation (47). Lu et al. (82) showed vertical shrinkage of isolated frog rod OSs in response to light stimulus, and transmission electron microscopy (TEM) observation confirmed shortened inter-disc spacing in light-adapted rod OSs. To better appreciate the physiological origin of the fast-photoreceptor IOS, comparative measurements of OS deformation and ERG were conducted, and it was consistently observed that the OS deformation occurs earlier than the onset of the ERG a-wave (83). Moreover, substituting a traditional superfusing medium with a low-sodium medium blocked the ERG a-wave response but preserved the stimulus-evoked rod OS deformations (83). In comparative photoreceptor-IOS recording and ERG measurement, previous studies confirmed that the response time of fast photoreceptor-IOS was ahead of the a-wave in the mouse retina (39, 62). This observation provides concrete evidence that the fast-photoreceptor IOS is independent of the OS hyperpolarization, i.e., cyclic guanosine monophosphate (cGMP) gated ion channel closure along the OS plasma membrane, the source of ERG a-wave. Instead, both the fast-photoreceptor IOS and rod OS deformations are associated with the early phase of the phototransduction cascade that involves the sequential activation of rhodopsin, transducin, and cGMP phosphodiesterase (PDE). A recent comparative study of wild-type (WT) and retinal degeneration 10 (rd10) mice demonstrated that fast photoreceptor-IOS occurs even earlier than PDE activation (84). Similarly, recent phase-resolved OCT imaging revealed a stimulus-evoked rapid reduction of OPL in photoreceptor OSs in the human retinas (32, 57, 69). The rapid OPL decrease showed a time course of millisecond level, which is in keeping with the earlier results of stimulus-evoked fast photoreceptor-IOS in animal models (45). Boyle et al. (58) suggested that contraction of the photoreceptor OS may be driven by the charge transfer across the OS disc membrane relevant to early receptor potential (ERP) (32), a fast electrical signal observed in cone photoreceptors under intense flash stimuli (85, 86). The ERP is associated with the conformational change of opsins embedded in the OS disc membrane and is distinct from the changes in the photoreceptor membrane potential.

**Figure 7:**
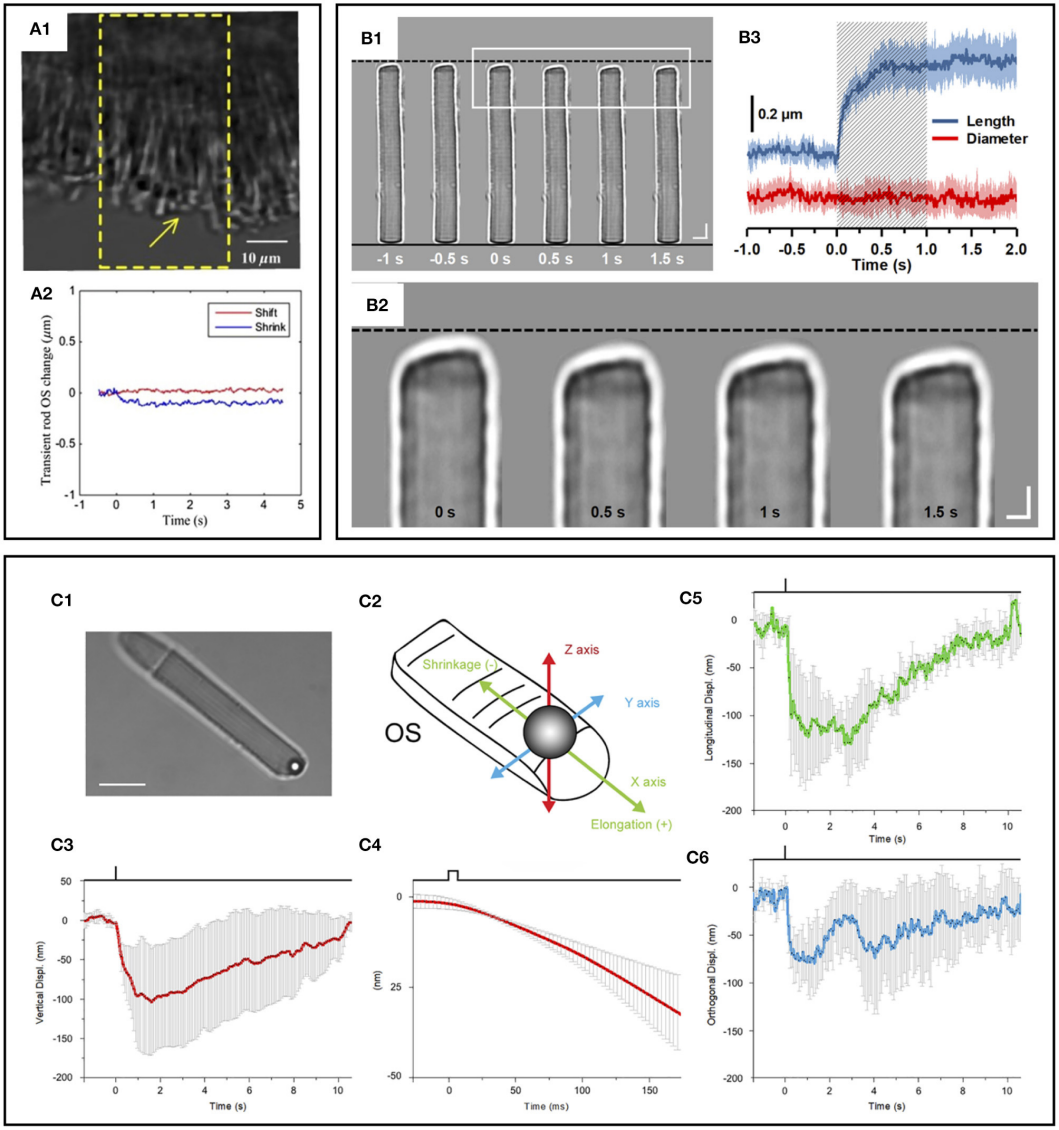
Photoreceptor outer segment (OS) shrinkage due to light stimulus. **(A1)** Stimulus-evoked mouse rod OS movement. The yellow window indicates the stimulation area. **(A2)** OS changes of a mouse rod photoreceptor. In the center of the stimulation region, the length of the OS shrunk, while in the peripheral region, the OS swung toward the center of the stimulation area in the plane perpendicular to the incident stimulus light. Reprinted with permission from Zhao et al. (88). **(B1)** Representative light microscopic images of an isolated frog rod OS acquired with an interval of 0.5 s. To better show the light-evoked OS shrinkage, the base of the rod OS in each image is aligned horizontally as shown by the black solid line at the bottom. The black-dashed line at the top represents the position of the rod OS tip at time −1 s. Scale bars (in white) represent 5 μm. **(B2)** Enlarged picture of the white rectangle in **(B1)**. Scale bars (in white) represent 2 μm. **(B3)** Time course of the averaged rod OS shrinkage in both length and diameter acquired from eight different rod OSs. Colored areas accompanying the curves represent the standard deviations. Shaded area indicates the 1-second stimulation period. Reprinted with permission from Lu et al. (82). **(C)** Mechanical response of an X. laevis rod to light flashes. The position of a bead sealed against the tip of the rod OS is monitored with optical tweezers. Following a bright flash of 491 nm, equivalent to about 10^4^ photoisomerization [R*], a transient shrinkage is observed. **(C1)** Bright-field infra-red image, showing a trapped bead in contact with the tip of the rod OS (scale bar, 10 μm). **(C2)** Detail of the 3D tracking system. **(C3)** Light-induced shifts in the Z axis of the trapped bead (downward is negative). **(C4)** Expansion of the time base in C5 to examine the delay between light stimulus and bead movement. **(C5)** Bead displacement along the direction of the rod OS (shrinkage is negative, and elongation is positive). **(C6)** Bead displacement in the direction perpendicular to the rod OS axis. Data are representative of mean ± SD of 5 different experiments. Reprinted with permission from Bocchero et al. (89).

Intriguingly, phase-resolved OCT imaging revealed not only a rapid (<5 ms) reduction in OPL after the stimulus onset but also a slower (>1 s) increase in OPL of the photoreceptor OSs (31, 32, 57, 69). The elongation response has been consistently observed in phase-resolved ORG studies. Zhang et al. (69) showed that the magnitude of these path length increments was positively correlated with stimulating light dose, and they used the photoreceptor elongation signals to generate maps of the three cone classes. It has been hypothesized that the increase (up to hundreds of nanometers) in the OPL of photoreceptor OSs after light stimulus would be attributable to osmotic swelling, an increase in the cytoplasmic volume due to excess osmolytes produced by phototransduction (66). Based on the intensity-based processing, Lu et al. (87) also observed photoreceptor OS elongation following intense light exposure and subsequent recovery, i.e., photoreceptor OS shortening, in human subjects. Although the increased OPL between IS/OS junction and OS tip is consistent among *in vivo* phase-resolved ORG measurements, there is a lack of direct evidence of the OS elongation. In fact, *ex vivo* studies have often shown conflicting results (Figures 7A–C). Comparative TEM observation disclosed shortened inter-disc spacing in rod OSs in light-adapted frog retinas (82). Fast X-ray diffraction studies also revealed a light-induced shrinkage of the disc lattice distance from the frog and mouse rod OSs (90, 91). Moreover, Bocchero et al. (89) recently measured light-evoked 3-axis (X, Y, Z plane) volume changes in the single rod OS from the Xenopus retina. They consistently observed a shortening of the OS on the order of 100–200 nm after a brief flash stimulus. The shortening was transient, and the OS returned to its original size within about 10 s, without further expansion.

Taken together, there is a growing consensus on the photoreceptor OS shrinkage at the early stage of phototransduction. However, more research is necessary to verify the OS swelling or relaxation mechanism. Note that the different dynamics between retinal explant and isolated single photoreceptor responses were demonstrated (90), and fundamentally, photoreceptor morphology and cellular compartment are different among species (92, 93).

### Transient Reflectance Changes in Inner Segment Ellipsoid Zone

**Figure 8:**
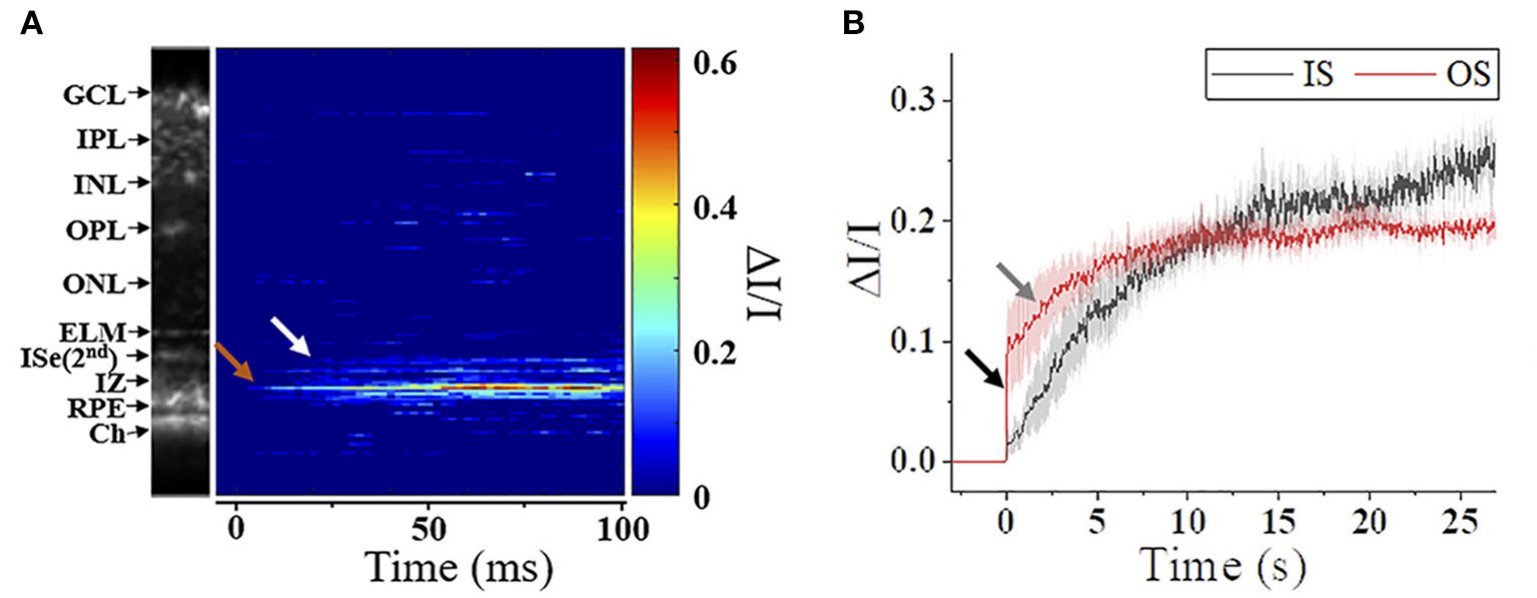
Metabolic response of photoreceptor inner segment. **(A)** IOS M-scan within 100 ms, the white and red arrowheads show the onsets of IS-IOS and OS-IOS, respectively. **(B)** Average IS-IOS and OS-IOS from six mice. The average OS-IOS showed a biphasic curve. The dark and gray arrowheads show the first rapidly increasing phase and the second gradually increasing phase, respectively. GCL, ganglion cell layer; IPL, inner plexiform layer; INL, inner nuclear layer; OPL, outer plexiform layer; ONL, outer nuclear layer; ELM, external limiting membrane; ISe, inner segment ellipsoid; IZ, interdigitation zone; RPE, retinal pigment epithelium; Ch, Choroid; IS, photoreceptor inner segment; OS, photoreceptor outer segment. Reprinted with permission from Ma et al. (61).

The photoreceptor ISe is the center of metabolism, consisting of abundant mitochondria (94). Despite contradictory nomenclatures, the ISe is a biomarker of photoreceptor integrity. The integrity of the ISe band has been correlated with different aspects of retinal function (95, 96). In addition, ISe reflectivity has recently emerged as a sensitive biomarker of photoreceptor structure due to the ISe reflectance changes under degenerative retinal conditions (96). In addition, a recent IOS recording showed that the ISe reflectivity dynamically changed in response to different light conditions (61, 65, 66). Figure 8 shows the ISe IOS in the mouse retina. A study demonstrated that stimulus-evoked IOS change at the ISe appeared with a time delay of ~12 ms after the stimulation, which is rather slower than fast-OS response, suggesting that the slow ISe IOS might reflect the metabolic reaction of mitochondria, following the phototransduction in the OS (61). Under metabolic stresses, the morphological structure and motion dynamics of mitochondria are all varied (97, 98), which could alter the optical signal properties of the ISe zone, resulting in OCT reflectance changes. In addition, Kim et al. recently found a significant reduction in ISe reflectance during dark adaptation in the mouse retina. This observation further emphasizes light-modulated ISe reflectivity could serve as a sensitive biomarker for photoreceptor dysfunction (65). However, Zhang et al. (66) hypothesized that phototransduction reactions associated with complete activation of G-protein alpha-subunit transducin might induce osmotic swelling, and the swelling, combined with the mass redistribution of transducing proteins from the disc membranes into the cytosol, might underlie the scattering increases at the IS/OS and OS tips. There is no doubt that the ISe reflectivity can be actively regulated under different light conditions, and it would be a potent biomarker for photoreceptor dysfunction. However, the ISe IOS source is still ill-defined, and both mitochondrial metabolic activity and redistribution of G proteins could simultaneously affect the signal. In addition, there is an unmet need to resolve ambiguity as to how well ISe reflectivity correlates with underlying photoreceptor structure (56).

### Subretinal Space Changes Under Different Light Conditions

The SRS is the extracellular fluid space between the ELM and the apical RPE, which is isolated by tight junctions at these two borders, and the ELM and RPE appear as hyper-reflective bands in OCT, which facilitates band change analysis. In fact, the SRS has been a well-recognized region that actively deforms under different light conditions (99). Thus, SRS dynamics could become a potential biomarker for outer retinal dysfunction.

**Figure 9:**
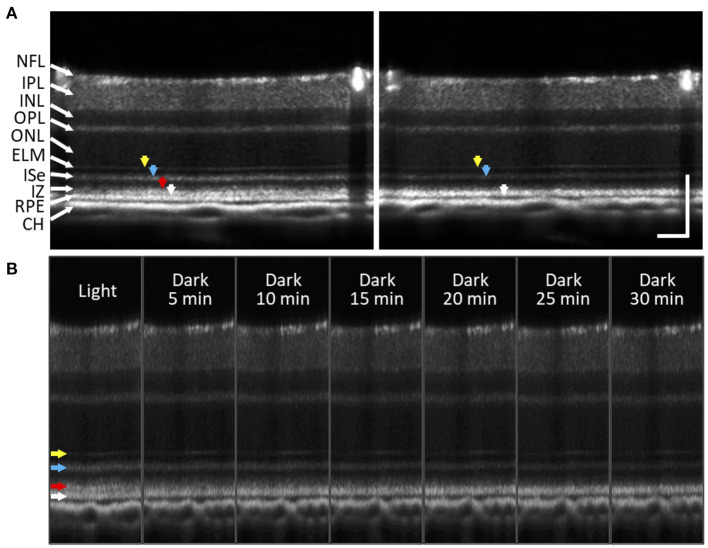
Subretinal space changes during dark adaptation in the mouse retina. **(A)** OCT images of light- and dark-adapted retina of a two-month-old C57BL/6J mouse. Color arrows to indicate outer retinal bands: 1st ELM band (yellow), 2nd ISe band (blue), 3rd IZ and OS tip band (red), 4th RPE band (white). **(B)** A sequence of OCT images obtained every 5 min up to 30 min during dark adaptation. During dark adaptation, ISe intensity reduction rapidly occurred, and the SRS became thinner. In addition, the 3rd outer retinal band (red arrow) faded over time. NFL, nerve fiber layer; IPL, inner plexiform layer; INL, inner nuclear layer; OPL, outer plexiform layer; ONL, outer nuclear layer; ELM, external limiting membrane; ISe, inner segment ellipsoid; IZ, interdigitation zone; RPE, retinal pigment epithelium; CH, choroid. Scale bars: 100 μm. Modified with permission from Kim et al. (65).

Using Fourier-domain OCT with intensity-based processing, Li et al. (100) found a notable thinning of the outer retina in dark-adapted mouse eyes. They further demonstrated that volume changes in the outer retina varied with the different stages of retinal degeneration in the rd10 mouse model (67). Berkowitz et al. (101) observed that a light-driven expansion of the outer retina was more distinguished in C57BL/6 mice than 129S6/SvEvTac mice. In addition, Gao et al. (102) recently demonstrated a significant reduction of the magnitude and width of a hypo-reflective band between the photoreceptor OS and RPE in the dark-adapted mouse and human retina. Lu et al. (87) also showed a rapid decline in the IS/OS-RPE distance after a light stimulus. Figure 9 illustrates the dark adaptation effects on the mouse retina. Kim et al. (65) recently demonstrated the dynamic SRS thinning of the mouse retina during light to dark transitional moment and found that dark-induced retinal response was reflected by transient structural (i.e., the SRS thinning) and physiological (i.e., ISe intensity reduction) changes. High-speed OCT recording further identified a strong correlation between the SRS thinning and ISe intensity reduction in the outer retina (65). The SRS thinning mechanism is understood by RPE-mediated water transport from the SRS. The light to dark transition is accompanied by an increase in photoreceptor metabolism, leading to increased oxygen consumption in the retina (103), which can acidify the outer retina due to increased CO_2_ and wastewater production, and eventually upregulate water removal co-transporters in the RPE. This removal of the acidified water has been linked to the SRS thinning (99, 104).

However, human study has sometimes shown intriguing but puzzling results. Messner et al. (105) observed a distance decrease between IS/OS junction and RPE after the light stimulus. They observed that the OS tips and RPE were drawn closer together after light exposure, speculating that this might be associated with a decrease in volume of the SRS (105), which is conflicting with previous observations (99). In addition, Azimipour et al. observed that the RPE band appears to split, with its apical portion moving toward the cone OS tip after the light stimulus. They speculated that light-driven translocation of melanin observed in amphibians (49) or a consequence of inward water movement across the RPE/Bruch's complex could be a potential signal source (106). In the human retina, photoreceptor cell population, rod/cone ratio, photoreceptor length, and morphology are largely different depending on eccentricity. The appearance of outer retinal bands also varies among different OCT systems (56). Thus, various factors that can potentially affect ORG measurement should be carefully accounted for and documented.

## Discussion

### Emerging Issues in ORG Measurements

As demonstrated above, outer retinal structures including the photoreceptor IS and OS, SRS, and RPE have been highlighted in ORG recording because of their structural clarity in OCT and importance as a primary target of retinal degenerative diseases. However, ORG recording has been conducted by different OCT systems and experimental protocols. Thus, there are often challenges for quantitative analysis using OCT reflectance information. Lee et al. (96) recently provided a valuable discussion about challenges associated with ISe intensity measurement using clinical OCT. They emphasized the importance of the pupil entry position of the OCT beam due to the altered reflectivity profile of the retinal image (107, 108). The reproducibility of ISe reflectivity measurements largely depends on the standardization of pupil entry point acquisition. There is also the importance of using appropriate intensity scales. Clinical OCT images are generally presented on a logarithmic scale, but this can misrepresent fundamental differences in reflectivity and a loss of information (109, 110). Adjusting intensity values can result in broadened hyper-reflective retinal bands, and accordingly vertical position of the hyper-reflective bands can be altered within the scan (109). Another issue is inter-device variation in ISe intensity, as each OCT device has different acquisition and optimization methods (111). Thus, it is necessary to establish a standardized normalization method (96). Meleppat et al. further demonstrated that the reflectivity from the inner retinal layers and ELM are also highly directional. The reflectance declines sharply with the angle of incidence of the OCT beam on the mouse retina (112). Notably, in albino mice, the reflection from Bruch's membrane was highly directional as well (112).

Aside from the issues of image acquisition and analysis, the unmet need for advancing ORG measurement is to establish anatomic correlation with underlying photoreceptor structure. Yao et al. (56) recently provided a valuable discussion about the interpretation of anatomic correlates of outer retinal bands in OCT. Clinical OCT, laboratory prototype OCT without AO, or AO-OCT can resolve four distinct hyperreflective bands in the outer retina (Figure 1C). Recent resolution improvement allows further separation of the RPE/Bruch's membrane complex into the individual layers (76). However, our understanding of anatomical correlates to each outer retinal band is not keeping pace with the recent development of OCT imaging technology. Understanding anatomical correlates to OCT bands is crucial to translate ORG information for diagnostic purposes. The interpretations of the 1^st^ ELM and 4^th^ RPE/Bruch's membrane of the outer retina are consistent; however, the interpretations of 2^nd^ band of ISe or IS/OS junction and 3^rd^ band of interdigitation zone (IZ) or OS tips remain a great controversy (109, 113, 114). The inconsistency is mainly found between clinical OCT and AO-OCT images. Comparative alignment of the outer retinal OCT bands with a scale model of outer retinal morphology showed the ISe and IZ as the correlates to the 2^nd^ and 3^rd^ outer retinal bands (115). The 2014 International OCT Nomenclature Meeting also affirmed the ISe and IZ as the anatomic correlates of the 2^nd^ and 3^rd^ bands of the outer retina (116). However, with improved spatial resolution, AO-OCT revealed a much thinner 2^nd^ outer retinal band than the ISe band observed in clinical OCT (117). AO-OCT measurement demonstrated that the 2^nd^ band thickness was about 4.7 μm (117), while the 2^nd^ band thickness in clinical OCT was about 16–20 μm (56). In addition, AO-OCT illustrated that the 2^nd^ band peak is closer to the 3^rd^ band peak, relative to the 1^st^ band peak. Taken together, it was suggested that the 2^nd^ band in AO-OCT corresponds to the IS/OS junction rather than the ISe. A similar phenomenon was also found in the 3^rd^ outer retinal band. The 3^rd^ band thickness was 4.3–6.4 μm and 14–19 μm in AO-OCT and clinical OCT, respectively (118). Yao et al. described that the AO-OCT might enhance the sensitivity for imaging ballistic photons from the IS/OS junction while partially rejecting the diffusive photons within the ISe region due to better sectioning capability (56). Thus, in clinical OCT, both IS/OS junction and ISe can non-exclusively contribute to the 2^nd^ band; and OS tips and RPE apical processes can simultaneously contribute to the 3^rd^ band. Also, weighting factors including system resolution, effective pupil size, imaging orientation, and aberrations can differentially affect the signal detection in clinical OCT and AO-OCT. Moreover, the OCT band profile is also known to be affected by optical dispersion (119). Therefore, it should be acknowledged that the contributing factors for individual band correlates are variable in different instruments, testing protocols, and eye conditions.

### Future Perspectives

Growing evidence indicates that morphological examination may be limited to detecting the early stage of retinal degenerative diseases, including but not limited to AMD (120), DR (121), and IRDs (122). Given that timely management of the diseases is the key to the preservation of vision (123–126), functional assessment of retinal photoreceptors and neurovascular coupling has gained increasing importance. Ongoing development in OCT-based ORG measurement is one of the most promising methodologies for screening people at risk. With unparalleled depth-resolved capability, recent advances in OCT further provide multi-modalities, ultrahigh-speed recording, single-cell resolution, and ultrawide field recording. Moreover, advanced ORG processing algorithms allow the mapping of various functional activities over morphological images. While the implementation of OCT-ORG in clinics is still at an early stage, recent studies demonstrated the feasibility of ORG measurement in human subjects (25, 31, 32, 36, 37, 54, 57, 69, 70, 102, 105). To facilitate clinical transition, it would be necessary to refine experimental procedures and shorten the examination time, including the light/dark adaptation to reduce the subject's burden. In addition, there is a broad range of existing testing methods for functional examination, and each has benefits and limitations. In this regard, OCT-ORG should be conducted alongside the existing tests as a multimodal evaluation, which can provide a better understanding of retinal physiology and corresponding IOS sources. Standardized imaging protocol and processing methods also need to be established as there are significant variations in imaging quality, the appearance of the retina, and following results due to different systems and processing algorithms. Above all, our understanding of functional activity and the corresponding IOS is quite limited. Thus, there is an unmet need to seek direct evidence of biological processes to visual stimulus, which helps translate distinct intrinsic signal sources at different retinal locations to target retinal disorders. Both *in vivo* and *ex vivo* studies using mutant animal models would be helpful. We anticipate that further development of the OCT system and ORG processing methods promises an objective measurement of neural and hemodynamic dysfunctions in the retina, allowing early detection and therapeutic assessment of AMD, DR, IRDs, and other retinal diseases.

## Author Contributions

T-HK conceived the article, performed the literature search, drafted the manuscript, and prepared figures. GM and TS edited the manuscript and prepared figures. XY conceived the article, edited the manuscript, and supervised the study. All authors approved the submitted version.

## Funding

National Eye Institute: P30 EY001792, R01 EY030101, R01 EY023522, R01 EY029673, R01 EY030842, and R44 EY028786; Richard and Loan Hill endowment; and unrestricted grant from Research to Prevent Blindness.

## Conflict of Interest

The authors declare that the research was conducted in the absence of any commercial or financial relationships that could be construed as a potential conflict of interest.

## Publisher's Note

All claims expressed in this article are solely those of the authors and do not necessarily represent those of their affiliated organizations, or those of the publisher, the editors and the reviewers. Any product that may be evaluated in this article, or claim that may be made by its manufacturer, is not guaranteed or endorsed by the publisher.
